# Twelve-week combined arginine and fish oil supplementation is associated with reduced sarcopenia severity: a randomized, double-blind, placebo-controlled study

**DOI:** 10.3389/fnut.2026.1763219

**Published:** 2026-03-02

**Authors:** Weixia Yuan, Panpan Ao, Fengfu Wu, Facui Xu, Ying Ma, Yun Ma, Shaofeng Wei, Lijia Yuan

**Affiliations:** 1 Department of Nutrition, Joint Logistics Support Force 925th Hospital, Guiyang, China; 2 School of Public Health, Guizhou Medical University, Guiyang, China

**Keywords:** aging, arginine, factors, fish oil, inflammatory, randomized controlled trial, sarcopenia

## Abstract

**Background:**

Sarcopenia is a geriatric syndrome characterized by the gradual loss of skeletal muscle mass and function, posing a major public health concern due to its link to various negative health outcomes. While supplementation with arginine or fish oil has shown potential in reducing muscle loss and functional decline, the combined effects of arginine and fish oil supplementation in sarcopenia remain largely unexplored.

**Objective:**

This study aimed to assess the impact of combining arginine and fish oil supplements on muscle strength, physical performance, body composition, and inflammation markers in older adults with sarcopenia.

**Methods:**

A 12-week randomized clinical trial was conducted from December 2023 to October 2024 with 29 older adults diagnosed with sarcopenia. Participants were randomly assigned to either the intervention group, which received arginine (14 g/day) and fish oil (6 g/day), or the control group, which received a placebo. The main outcomes measured were handgrip strength, gait speed, 5-time chair stand test, and skeletal muscle index (SMI). Secondary outcomes included inflammatory markers, nutritional status, blood lipid levels, frailty, physical activity, and sleep quality. Statistical analyses involved multiple comparisons to evaluate differences between the groups.

**Results:**

At the end of the 12-week trial, gait speed significantly increased in the Arg + fish oil groups (*p* < 0.05), whereas no significant change was observed in the placebo group. Furthermore, gait speed and grip strength in the Arg + fish oil group were significantly higher than in the placebo group (*p* < 0.05). Regarding inflammatory markers, compared with baseline, the placebo group showed a significant increase in TNF-α, while the Arg + fish oil group showed a significant decrease in IL-6 (*p* < 0.05). Moreover, TNF-α and IL-6 levels in the Arg + fish group were significantly lower than those in the placebo group (*p* < 0.05). Quality of life assessments revealed that the Arg + fish group demonstrated significantly greater improvement in PASE and frailty compared to the placebo group.

**Conclusion:**

Supplementing with both arginine and fish oil significantly boosted muscle strength, physical performance, and reduced levels of inflammation in older adults with sarcopenia. These initial findings suggest that this approach could be a potential strategy for managing sarcopenia by improving muscle function and lowering systemic inflammation.

## 1 Introduction

Sarcopenia, a geriatric syndrome characterized by progressive loss of skeletal muscle mass, diminished muscle strength, or impaired physical performance, has emerged as a significant public health concern in aging populations (1). This condition is strongly associated with functional decline and increased disability risk, substantially impacting healthcare systems worldwide. Epidemiological studies indicate that the prevalence of sarcopenia among older Asian populations ranges from 6.8 to 25.7%, underscoring its clinical significance (2). The pathogenesis of sarcopenia is multifactorial, involving complex interactions between age-related physiological changes and molecular mechanisms (3, 4). Key contributing factors include the downregulation of anabolic hormones (insulin, sex steroids, and growth hormone), increased muscle fiber apoptosis, and elevated circulating levels of pro-inflammatory cytokines (5–8). Given the pivotal role of chronic inflammation and anabolic-catabolic imbalance in sarcopenia progression, therapeutic strategies targeting these pathways, particularly through immunonutrition interventions (e.g., arginine and omega-3 fatty acids from fish oil), have shown promising potential in clinical research.

Immunonutrition supplementation (particularly with L-arginine (L-Arg), a conditionally essential amino acid with significant anabolic properties in muscle metabolism) has demonstrated efficacy in improving muscle mass and function. The therapeutic mechanisms of L-Arg are primarily mediated through two pathways: (1) its metabolite, creatine, activates the PI3K-Akt/PKB-mTOR signaling pathway to enhance muscle protein synthesis (9), and (2) its conversion to nitric oxide (NO) stimulates satellite cell proliferation and increases satellite cell numbers in muscle fibers, thereby restoring skeletal muscle function (10). Similarly, fish oil, a rich source of n-3 polyunsaturated fatty acids (PUFAs), has shown significant potential as a nutritional intervention for sarcopenia. The n-3 PUFAs exert their effects through multiple mechanisms: reducing plasma levels of pro-inflammatory cytokines (e.g., IL-6 and TNF-α), increasing anti-inflammatory factors (e.g., IL-10 and TGF-β) (11), and stimulating the mTOR signaling pathway to enhance muscle protein synthesis while inhibiting protein degradation (12–14).

Emerging evidence suggests a synergistic relationship between fish oil and arginine, with studies demonstrating that fish oil can enhance arginine bioavailability and activity (15, 16). Extensive clinical research has established the beneficial effects of arginine and fish oil supplementation in various pathological conditions, including cancer, gastrointestinal disorders, renal diseases, critical illness, and cerebrovascular accidents (17). Furthermore, numerous clinical trials and meta-analyses have consistently demonstrated the efficacy of combined L-arginine and fish oil formulations used in surgical populations (18–21).

Notably, plasma levels of arginine and n-3 PUFAs decrease with age (22, 23), which provides a strong rationale for supplementation in older adults. While many studies have explored the effects of arginine or fish oil supplements individually in patients with sarcopenia, there is a significant gap in research on their combined therapeutic potential.

This study aims to assess the effectiveness of combining arginine and fish oil supplementation in improving muscle strength, physical function, and body composition in older adults with sarcopenia, and examine the impact of this intervention on inflammatory markers, nutritional status, and other relevant health factors. We hypothesize that the combined supplementation will significantly improve muscle function and quality of life in patients with sarcopenia through two key mechanisms: (1) modulating inflammatory pathways and (2) boosting muscle protein synthesis. To our knowledge, this is the first to comprehensively explore the combined effects of arginine and fish oil supplementation in sarcopenia patients, which could help develop new multi-target strategies for managing this debilitating condition.

## 2 Materials and methods

### 2.1 Study design

The present study was a randomized (1:1), controlled trial of newly diagnosed with Sarcopenia over 60 years of age. Participants were selected and recruited from the 925th Hospital in China. This study was conducted according to the guidelines laid down in the Declaration of Helsinki and all procedures involving human patients were approved by the ethics committee of the Joint Logistics Support Force 925th Hospital; ethics number: YNKT20230601. The study procedures and protocol were approved and registered with the Chinese Clinical Trial Registry (ChiCTR2300078053). All patients provided written informed consent to participate in this study.

The sample size estimation of this study is based on previous research on the 6-meter gait speed of elderly patients with sarcopenia in Guizhou Province. It was calculated that each group had at least 22 participants to ensure 80% power (24).

### 2.2 Selection of participants in the study

Eligible participants were selected and recruited from the hospital, based on sarcopenia in the Chinese Expert Consensus on Prevention and Control of sarcopenia in older adults (2023). Participants were screened using specific inclusion and exclusion criteria. The inclusion criteria were as follows: newly diagnosed with sarcopenia, aged 60–100 years. Exclusion criteria: (1) patients with severe cognitive impairment and mental illness unable to take the test; (2) patients with metal implants; (3) patients with severe dysfunction of heart, liver, kidney, and other organs; (4) patients with edema; (5) patients with severe sepsis or systemic inflammatory reaction syndrome; (6) bedridden patients and using enteral nutrition.

### 2.3 Intervention and randomization

This study was a double-blind, placebo-controlled, randomized trial. Eligible subjects were randomly assigned in a 1:1 ratio to either the combined intervention group or the placebo group. The randomization numbers were conducted using a computer-generated randomization. Once they were included (study day 0), participants were randomly assigned using a computer-generated list (1:1 block randomization) to either the intervention (Arg + fish oil) or the control group (placebo). The Sealed Envelope method was employed for randomization, in which each code was sealed in opaque envelopes and numbered sequentially. A nurse who was not directly involved in the study was asked to open each envelope sequentially to distribute arginine, fish oil, and placebo as needed. Subjects, nurses distributing the reagents, and the study investigators (responsible for outcome assessment) all remained blinded to the group assignments.

Participants in the Arg + fish oil group received daily 14 g arginine and 6 soft gel capsules (DHA 1.26 g, EPA 1.92 g), respectively. Each fish oil capsule (BY-HEALTH, Chain) contained 320 mg Eicosapentaenoic acid (EPA) and 210 mg Docosahexaenoic acid (DHA), arginine was manufactured and supplied by the Huaheng Biotechnology Co., Ltd. Whereas participants in the control group were given a placebo with a trait similar to arginine (Sugar free lotus root powder 14 g, energy: 52 kcal, carbohydrate: 12.8 g, protein: 0 g, fat: 0 g). Except for different intervention substances, two groups received the same dietary and exercise education guidance.

The patients were monitored regularly by the first researcher through regular phone calls and messages to ensure compliance. Compliance was evaluated by the total capsule or arginine count every week during the patients’ hospital visits through a meticulous count of the tablets. All data were collected from participants at baseline (study day 0) and at the end of the completed trial at 12 weeks as end-trial. In addition, safety assessments included adverse events that occurred during the reporting period.

### 2.4 Outcome measures

The primary outcome was muscle condition assessed by muscle-related measures (grip strength, 5-time chair stand test, 6-meter gait speed test, skeletal muscle mass index (SMI), calf circumference, while the secondary outcome was inflammatory factors, nutritional status, blood lipid, frailty, physical activity, and sleep. Various study procedures were employed, including face-to-face interviews, anthropometric measurements, and blood collection.

### 2.5 General socio-demographic characteristics and anthropometric assessments

A face-to-face interview was conducted based on a structured questionnaire to assess the socio-economic and demographic profile, dietary patterns, and lifestyle-related behavioral practices. In addition, information on personal medical history, residence status, illness, diet expenses, marital status, living area, smoking and drinking status.

#### 2.5.1 The physical activity scale for the elderly

The Physical Activity Scale for the Elderly (PASE) is a brief (5-min) and easily scored survey designed specifically to assess physical activity in epidemiologic studies of persons aged 65 years and older (25). The PASE score combines information on leisure, household, and occupational activity. The total PASE score was computed by multiplying the amount of time spent in each activity (hours/week) or participation (yes/no) in an activity by the empirically derived item weights and summing over all activities.

#### 2.5.2 FRAIL questionnaire

The FRAIL (26) scale includes 5 components: Fatigue, Resistance, Ambulation, Illness, and Loss of weight. Frail scale scores range from 0 to 5 and represent frail (3., 4., 5.), pre-frail (1., 2.), and robust (0) health status. Fatigue was measured by asking respondents how much time during the past 4 weeks they felt tired with responses of “all of the time” or “most of the time” scored 1 point. Resistance was assessed by asking participants if they had any difficulty walking up 10 steps alone without resting and without aids, and Ambulation by asking if they had any difficulty walking several hundred yards alone and without aids; “yes” responses were each scored as 1 point. Illness was scored 1 for respondents who reported 5 or more illnesses out of 11 total illnesses. Loss of weight was scored 1 for respondents with a weight decline of 5% or greater within the past 12 months based on self-report.

#### 2.5.3 Pittsburgh sleep quality index

Participants completed the Pittsburgh Sleep Quality Index (PSQI) (27). A global score and seven component scores can be derived from the scale. The component scores are the following: Subjective sleep quality, sleep latency, sleep duration, sleep efficiency, sleep disturbances, use of sleeping medications and daytime dysfunction. Each component is scored on a scale from 0 to 3, with the total score ranging from 0 to 21; where a higher score describes poorer sleep quality.

#### 2.5.4 Nutritional status

Nutritional status was assessed by Short-Form Mini-Nutritional Assessment (MNA-SF), Nutrition Risk Screening 2002 (NRS2002) and waist circumference.

The MNA-SF (28) tool consists of 6 parameters: weight loss, appetite, mobility, psychological stress, neuro-psychological problems and body mass index (BMI). All parameters scored from zero to two or three with a total score of 0–14 thus classifying patients into 3 nutritional groups: well-nourished, at risk of malnutrition and malnourished if scores of 12–14, 8–11, and 0–7 points, respectively, were received.

The NRS2002 contains the following parameters (29): BMI, recent weight loss, recent decrease in food intake and severity of illnesses. Patients are scored by two components: nutrition and disease severity; a score of 0–3 according to whether these components are absent, mild, moderate or severe. The total score ranges from 0 to 6. Patients who were nutritionally at risk received a score of 3 or higher. Moreover, one point was added for a person > 70 years old.

#### 2.5.5 Dietary intake

According to the results of the questions and pre-experiment, the simple food-frequency questionnaire (FFQ 25) was selected for the dietary survey (30). By investigating the usual frequency of consumption and the amount consumed each time of specific foods over the past year in patients. Then, calculate the daily intake of energy and other nutrients based on the food composition table. In the statistical analysis of nutrient intakes, we excluded total energy intake if it fell below 300 kcal or exceeded 5,000 kcal, as these values were considered implausible.

#### 2.5.6 Biochemical indicators

5 ml of fasting venous blood was collected from all patients, and the serum was separated by centrifugation at a centrifugation radius of 10 cm and a centrifugation rate of 3,000 r/min for 10 min. The liver function (prealbumin, globulin, albumin, total protein et al), electrolyte levels (magnesium, calcium, chloride, potassium, sodium), and renal function (Uric acid, Creatinine, Carbamide) were analyzed using a blood biochemical analyzer (Hitachi 7060 automatic Clinical Analyzer, Japan). Hemoglobin and lymphocyte percentage were detected by blood cell analyzer, liver function, and renal function by serum enzyme method.

The levels of TNF-α, IFN-γ, IL-17, IL-10, IL-6, IL-4, and IL-2 were measured using a seven-cytokine detection kit (flow fluorescence luminescence method) (Hunan Weigong Biotechnology Co., Ltd., Hunan, China).

#### 2.5.7 Physical examination

Body weight was measured to an accuracy of 0.1 kg using a body composition analyzer (Model: Inbody 770, Korea), while height was assessed to the nearest 0.5 cm in the standing position without shoes by using a stadiometer. Waist circumference was measured with a non-stretchable tape measure, and recorded to the nearest 0.1 cm. The measurement was taken horizontally around the abdomen at the level of the landmarked point, drawn at the uppermost lateral border of the iliac crest. For calf circumference, a non-stretchable measuring tape was looped horizontally around the calf. The tape was moved up and down until the greatest calf circumference was found, and the circumference was recorded to the nearest 0.1 cm. The evaluation of treatment safety was based on adverse events reported by the participants, and biochemical parameters (urea and creatinine) were measured at the baseline and at the end of the trial during the hospital visits.

#### 2.5.8 Statistical analysis

The normal distribution of variables was assessed and determined by the Kolmogorov–Smirnov test. If the *p*-value of the Kolmogorov–Smirnov test is larger than 0.05, then the data have a normal distribution and parametric statistical analysis, Student’s *t*-test, was conducted. Conversely, if the data do not follow a normal distribution (*p* < 0.05), non-parametric statistical analyses. Quantitative parameters were presented as mean ± standard deviation (SD), while categorical variables were shown as frequencies and proportions. Comparison of variables within the groups from baseline (baseline) and at the end of intervention (end-trial) was performed using sample *t*-tests for all continuous variables. The Bonferroni correction method was used for multiple comparison adjustment. All statistical analyses were performed using the SPSS (version 27.0) with statistical significance for all tests defined by the *p* < 0.05. A adjusted *p <* 0.017 indicates a statistically significant result.

## 3 Results

Supplementary Figure 1 shows the trial enrollment based on the CONSORT flow diagram. About 464 patients were screened in the present study; 113 were diagnosed with sarcopenia; 71 refused to participate because of remote residence, inconvenient transportation, refusal of venous blood collection, and private time schedule conflict with trial. Finally, a total of 42 eligible participants were recruited and included in the study. The recruitment of participants commenced on December 2023, and extended with follow-ups until the end of October 2024. Throughout the study, 13 participants dropped out due to several reasons such as non-compliance with supplements or three-quarters of the supplements were not consumed, refusal to provide blood sample at the end of the study, change of place of residence. Consequently, the final number included in the analyses was 29 participants.

### 3.1 General characteristics of the participants at baseline

Table 1 shows the baseline information for socio-demographic, dietary and lifestyle characteristics of the participants based on the experimental groups. In general, there were no significant differences in socio-demographic, dietary and lifestyle-related factors between these two groups.

**TABLE 1:** Demographic characteristics of the intervention group versus control group before interventions.

Index	Arg + fish oil(*n* = 14)	Placebo (*n* = 15)	t/Z/χ2	*p*
Age (x ± s, year)	80.00 ± 11.27	77.93 ± 7.29	0.590	0.560
**Gender [n(%)]**				
Male	9 (64.3)	7 (46.7)	0.909	0.340
Female	5 (35.7)	8 (53.3)		
**Race [n(%)]**				
Han nationality	13 (92.9)	13 (86.7)	0.299	1.000
Other	1 (7.1)	2 (13.3)		
**Living area [n(%)]**				
Urban	11 (78.6)	11 (73.3)	0.109	1.000
Rural	3 (21.4)	4 (26.7)		
**Dwelling state [n(%)]**				
Living alone	9 (64.3)	10 (66.7)	2.778	0.096
Other	5 (35.7)	5 (33.3)		
**Smoking status [n(%)]**				
Never smokers	8 (57.1)	9 (60.0)	0.024	0.988
Past smokers	5 (35.7)	5 (35.7)		
Current smokers	1 (7.1)	1 (14.0)		
**Drinking status**				
Never drinkers	8 (57.1)	10 (66.7)	3.692	0.158
Past drinkers	3 (21.4)	5 (33.33)		
Occasional drinkers	3 (21.4)	0 (0.00)		
**Drug types [n(%)]**				
≤ 5	9 (64.3)	5 (33.3)	2.778	0.096
>5	5 (35.7)	10 (66.7)		
**Hour of sleep [n(%)]**				
≤ 7 h	12 (85.7)	8 (53.3)	2.196	0.109
>7 h	2 (14.3)	7 (46.7)		
**Marital status [n(%)]**				
Married	12(85.7)	11(73.3)	0.132	0.651
Other	2(14.3)	4(26.7)		
**Education [n(%)]**				
No education	4 (28.6)	5 (33.3)	1.897	0.387
Middle or below	3 (21.4)	6 (40.0)		
Senior or above	7 (50.00)	4 (26.7)		
**Daily step count [n(%)]**				
≤ 6,000	9 (64.3)	9 (60.0)	0.056	0.812
>6,000	5 (35.7)	6 (40.0)		
**Monthly dietary expenses [n(%)]**				
< ¥500	3 (21.4)	4 (26.7)	0.843	0.656
¥500∼1,000	7 (50.0)	5 (33.3)		
> ¥1,000	4 (28.6)	6 (40.0)		
**Sickness expenses each year [n(%)]**				
<1 ¥000	1 (7.1)	1 (6.7)	0.299	0.861
¥1,000∼5,000	5 (35.7)	4 (26.7)		
> ¥5,000	8 (57.1)	10 (66.7)		
**Previous occupational physical labor intensity [n(%)]**				
Mild	6 (42.9)	7 (46.7)	0.042	0.979
Moderate	6 (42.9)	6 (40.0)		
Severe	2 (14.3)	2 (13.3)		

### 3.2 Dietary intakes of the participants

Table 2 shows the daily dietary intake of the participants according to the two groups. In the baseline, no significant differences were found for dietary intake such as daily intakes of energy, protein, total fat and carbohydrate intakes. In addition, no significant differences were found for the intake of arginine, DHA and EPA in the two groups.

**TABLE 2:** Comparison of dietary intake between baseline of the participants in the two groups.

Variables	Arg + fish oil (*n* = 14)	Placebo (*n* = 15)	t/z	*p*-Value
Grains (g/d)	228.68 ± 72.61	195.39 ± 74.34	1.219	0.233
Meat (g/d)	60.87 ± 23.54	66.81 ± 31.24	–0.603	0.552
Eggs (g/d)	49.69 ± 18.97	55.71 ± 83.89	–1.47	0.142
Milk (g/d)	176.34 ± 113.43	129.50 ± 143.72	–1.469	0.142
Legumes (g/d)	7.24 ± 5.57	6.71 ± 6.12	–0.153	0.878
Aquatic products (g/d)	9.04 ± 12.74	4.14 ± 7.34	–0.329	0.742
Vegetables (g/d)	290.16 ± 132.68	277.28 ± 154.89	–0.504	0.614
Fruits (g/d)	139.44 ± 63.81	95.09 ± 77.63	1.77	0.088
Oil (g/d)	25.23 ± 9.31	21.45 ± 12.26	–0.733	0.463
Energy (kcal/d)	1417.19 ± 349.34	1262.07 ± 325.97	1.246	0.223
Protein (g/d)	50.88 ± 13.82	47.95 ± 19.24	0.469	0.643
Total fat (g/d)	45.48 ± 12.88	40.50 ± 13.14	1.029	0.313
Carbohydrate (g/d)	205.60 ± 57.58	182.71 ± 68.85	1.038	0.308
Arginine (mg/d)	3050.45 ± 845.68	2870.60 ± 1174.12	–1.091	0.275
Total fatty acids (g/d)	56.09 ± 15.36	51.81 ± 16.78	0.683	0.501
Omega3, DHA (g/d)	0.18 ± 1.25	0.08 ± 3.15	–0.329	0.742
Omega3, EPA (g/d)	0.09 ± 0.13	0.04 ± 1.47	–0.329	0.742

### 3.3 Primary outcome

Table 3 presents the primary outcome, assessed by the 6-meter gait speed, 5-time chair stand, grip strength, SMI, and calf circumference of the participants based on two experimental groups. At baseline, there were no significant differences for the 6-meter gait speed, 5-time chair stand, grip strength, SMI, and calf circumference. Comparisons within the group at the end of the trial over 12 weeks showed a significant increment in the gait speed in the Arg + fish oil group from the baseline, suggesting a somatic function turns (*p* = 0.009). When the outcome of measurements was compared between intervention groups, it was found that the gait speed and grip strength were significantly higher in the Arg + fish oil group than in the placebo group at the end of the trial (*p* < 0.05).

**TABLE 3:** Comparison of primary outcome between baseline and end-line of the participants in the two groups.

Variables	Arg + fish oil (*n* = 14)	Placebo (*n* = 15)	*p*-value^b^	p-adjust^b^
**Gait speed (m/s)**				
Baseline	0.78 ± 0.36	0.81 ± 0.19	0.955	0.318
End of trial	1.23 ± 0.35	0.88 ± 0.23		
Change^c^	0.45 ± 0.39	0.07 ± 0.26	0.009	0.003
*p*-value^a^	0.009	0.333		
p-adjust^a^	0.003	0.111		
**5-time chair stand (s)**				
Baseline	14.18 ± 4.86	13.86 ± 3.44	0.996	0.332
End of trial	12.68 ± 3.77	14.35 ± 5.56		
Change^c^	–1.19 ± 3.90	0.49 ± 5.15	0.346	0.115
*p*-value^a^	0.494	0.776		
p-adjust^a^	0.165	0.259		
**Grip strength (kg)**				
Baseline	18.68 ± 4.99	18.36 ± 4.35	0.948	0.316
End of trial	20.96 ± 5.34	16.85 ± 3.77		
Change^c^	2.49 ± 3.30	–1.51 ± 3.08	0.003	0.001
*p*-value^a^	0.238	0.319		
p-adjust^a^	0.079	0.106		
**Calf circumference (cm)**				
Baseline	30.85 ± 1.34	29.44 ± 2.61	0.069	0.023
End of trial	30.67 ± 3.6	29.24 ± 2.39		
Change^c^	–0.25 ± 3.41	–0.20 ± 1.23	0.956	0.318
*p*-value^a^	0.819	0.832		
p-adjust^a^	0.273	0.277		
**SMI (kg/m^2^)**				
Baseline	5.75 ± 0.66	5.25 ± 0.83	0.083	0.028
End of trial	5.75 ± 0.91	5.37 ± 0.74		
Change^c^	–0.02 ± 0.89	0.12 ± 0.57	0.610	0.203
*p*-value^a^	0.952	0.667		
p-adjust^a^	0.317	0.222		

^a^Within-group differences; *p*-value is based on paired *t*-test. ^b^Comparison between groups; *p*-value is based on two independent sample *t*-test. ^c^Difference before and after the intervention. Mean difference represented. The p-adjust less than 0.017 indicates a statistically significant result.

### 3.4 Secondary outcome

Table 4 presents the Inflammatory conditions, assessed by the TNF-α, IFN-γ, IL-17, IL-10, IL-6, IL-4, IL-2 of the participants based on two experimental groups. At baseline, there were no significant differences for all inflammatory factors. Comparisons within the group at the end of the trial 12 weeks showed a significant increment in the TNF-αin the placebo group from the baseline (*p* = 0.025). On the contrary, participants in the Arg + fish oil group had significantly reduced the IL-6 at the end of the trial (*p* < 0.05). When the outcome of measurements was compared between intervention groups, it was found that the TNF-α and IL-6 were significantly lower in the Arg + fish oil group than in the placebo group at the end of the trial (*p* < 0.05).

**TABLE 4:** Comparison of inflammatory factors between baseline and end-line of the participants in the two groups.

Variables	Arg + fish oil (*n* = 14)	Placebo (*n* = 15)	*p*-value^b^	p-adjust^b^
**TNF-α(pg/mL)**				
Baseline	3.53 ± 2.02	2.61 ± 1.00	0.132	0.044
End of trial	2.38 ± 1.43	3.68 ± 1.43		
Change^c^	–1.15 ± 2.38	1.07 ± 1.28	0.004	0.014
*p*-value^a^	0.107	0.025		
p-adjust^a^	0.036	0.008		
**IFN-γ(pg/mL)**				
Baseline	12.43 ± 14.81	9.73 ± 11.76	0.596	0.200
End of trial	4.45 ± 2.57	4.76 ± 3.97		
Change^c^	–7.98 ± 15.38	–4.97 ± 12.73	0.576	0.192
*p*-value^a^	0.078	0.132		
p-adjust^a^	0.026	0.044		
**IL-17(pg/mL)**				
Baseline	2.42 ± 2.21	2.45 ± 3.08	0.974	0.324
End of trial	1.34 ± 0.90	1.65 ± 1.34		
Change^c^	–1.07 ± 2.35	–0.80 ± 2.83	0.785	0.262
*p*-value^a^	0.125	0.368		
p-adjust^a^	0.042	0.123		
**IL-10(pg/mL)**				
Baseline	9.98 ± 13.07	9.49 ± 12.43	0.920	0.307
End of trial	3.46 ± 1.28	2.93 ± 1.68		
Change^c^	–6.52 ± 13.47	–6.56 ± 13.01	0.994	0.331
*p*-value^a^	0.538	0.081		
p-adjust^a^	0.179	0.027		
**IL-6(pg/mL)**				
Baseline	7.46 ± 5.83	4.28 ± 1.95	0.081	0.027
End of trial	3.28 ± 1.66	4.45 ± 2.20		
Change^c^	–4.18 ± 5.79	0.16 ± 3.32	0.020	0.007
*p*-value^a^	0.026	0.831		
p-adjusta	0.009	0.277		
**IL-4(pg/mL)**				
Baseline	3.38 ± 4.67	2.20 ± 3.34	0.443	0.148
End of trial	8.04 ± 3.62	5.82 ± 7.17		
Change^c^	4.65 ± 5.25	3.62 ± 8.84	0.796	0.265
*p*-value^a^	0.009	0.101		
p-adjusta	0.003	0.034		
**IL-2(pg/mL)**				
Baseline	1.14 ± 1.68	0.85 ± 1.13	0.604	0.201
End of trial	0.37 ± 0.49	0.31 ± 0.46		
Change^c^	–0.77 ± 1.80	–0.54 ± 1.00	0.684	0.228
*p*-value^a^	0.136	0.100		
p-adjusta	0.045	0.033		

Interferon-γ: IFN-γ, Interferon-γ: IFN-γ. ^a^ Within-group differences; *p*-value is based on paired *t*-test. ^b^ Comparison between groups; *p*-value is based on two independent sample *t*-test. ^c^ Difference before and after the intervention. The mean difference represented. The p-adjust less than 0.017 indicates a statistically significant result.

Table 5 presents the PASE, PSQI, and FRAIL of the participants based on two experimental groups. At baseline, there were no significant differences for PASE, PSQI, and FRAIL. When the outcome of measurements was compared between intervention groups, it was found that the PASE and FRAIL were significantly improvement in the Arg + fish oil group than in the placebo group at the end of the trial (PASE: 5.96 ± 4.00 vs. 1.19 ± 6.30, *p* = 0.027; FRAIL: -0.64 ± 0.74 vs. 0.07 ± 0.80, *p* = 0.035).

**TABLE 5:** Comparison of PASE, PSQI, and FRAIL between baseline and end-line of the participants in the two groups.

Variables	Arg + fish oil (*n* = 14)	Placebo (*n* = 15)	*p*-value^b^	p-adjust^b^
**PASE**				
Baseline	36.65 ± 20.25	27.84 ± 18.84	0.244	0.081
End of trial	42.60 ± 20.28	29.03 ± 19.24		
Change^c^	5.96 ± 4.00	1.19 ± 6.30	0.027	0.009
*p*-value^a^	0.461	0.835		
p-adjust^a^	0.154	0.278		
**PSQI**				
Baseline	9.62 ± 3.10	10.40 ± 4.15	0.581	0.194
End of trial	10.62 ± 2.72	10.87 ± 3.76		
Change^c^	1.00 ± 2.31	0.47 ± 3.02	0.609	0.203
*p*-value^a^	0.391	0.467		
p-adjust^a^	0.130	0.156		
**FRAIL**				
Baseline	1.43 ± 1.02	1.53 ± 0.83	0.740	0.237
End of trial	0.79 ± 0.80	1.60 ± 0.83		
Change^c^	–0.64 ± 0.74	0.07 ± 0.80	0.035	0.012
*p*-value^a^	0.087	0.771		
p-adjust^a^	0.029	0.257		

^a^ Within-group differences; *p*-value is based on paired *t*-test. ^b^ Comparison between groups; *p*-value is based on two independent sample *t*-test. ^c^ Difference before and after the intervention. The mean difference represented. The p-adjust less than 0.017 indicates a statistically significant result.

Table 6 presents the nutritional Risk Status, assessed by the MNA-SF, NRS2002, and waist circumference of the participants based on two experimental groups. At baseline and the end of the trial there were no significant differences in nutritional risk status.

**TABLE 6:** Nutritional Risk Status was Assessed by the MNA-SF, NRS2002 and waist circumference of the Participants.

Variables	Arg + fish oil (*n* = 14)	Placebo (*n* = 15)	*p*-Value^b^	p-adjust^b^
**MNA-SF Scores**				
Baseline	10.29 ± 2.20	9.40 ± 2.10	0.234	0.078
End of trial	11.39 ± 1.82	10.38 ± 1.72		
Change^c^	1.16 ± 1.56	0.98 ± 1.92	0.876	0.292
*p*-value^a^	0.158	0.189		
p-adjust^a^	0.053	0.063		
**NRS2002 Scores**				
Baseline	2.21 ± 0.89	2.87 ± 1.06	0.085	0.028
End of trial	1.69 ± 0.48	2.68 ± 0.71		
Change^c^	–0.47 ± 0.66	–0.19 ± 1.16	0.398	0.133
*p*-value^a^	0.075	0.552		
p-adjust^a^	0.025	0.184		
**Waist circumference (cm)**				
Baseline	81.46 ± 9.56	75.55 ± 7.97	0.081	0.027
End of trial	79.37 ± 7.23	76.01 ± 8.54		
Change^c^	–2.09 ± 4.92	0.46 ± 2.42	0.085	0.028
*p*-value^a^	0.520	0.880		
p-adjust^a^	0.173	0.293		

^a^ Within-group differences; *p*-value is based on paired *t*-test. ^b^ Comparison between groups; *p*-value is based on two independent sample *t*-test. ^c^ Difference before and after the intervention. The mean difference is represented. The p-adjust less than 0.017 indicates a statistically significant result.

Table 7 presents the blood lipids level Assessed by the LDL, HDL, total cholesterol, and triglycerides of the participants based on two experimental groups. At baseline, there were no significant differences in blood lipid levels. When the blood lipids level was compared between intervention groups, it was found that the triglycerides were significantly improvement in the Arg + fish oil group than the placebo group at the end of the trial (–0.97 ± 0.81 vs. –0.12 ± 0.99, *p* = 0.022).

**TABLE 7:** Blood lipid levels were Assessed by the LDL, HDL, total cholesterol, and triglycerides of the participants.

Variables	Arg + fish oil (*n* = 14)	Placebo (*n* = 15)	*p*-value^b^	p-adjust^b^
**LDL (mmol/L)**				
Baseline	2.09 ± 0.68	2.05 ± 0.95	0.965	0.322
End of trial	2.09 ± 0.83	2.48 ± 0.72		
Change^c^	0.06 ± 1.29	0.43 ± 1.21	0.436	0.145
*p*-value^a^	0.853	0.176		
p-adjust^a^	0.284	0.059		
**HDL (mmol/L)**				
Baseline	1.47 ± 0.41	1.45 ± 0.36	0.825	0.275
End of trial	1.60 ± 0.46	1.51 ± 0.63		
Change^c^	0.18 ± 0.36	0.05 ± 0.60	0.515	0.172
*p*-value^a^	0.293	0.773		
p-adjust^a^	0.098	0.258		
**Total cholesterol (mmol/L)**				
Baseline	4.2 ± 1.02	4.03 ± 1.21	0.871	0.290
End of trial	3.79 ± 1.32	4.15 ± 0.95		
Change^c^	–0.30 ± 1.44	0.13 ± 1.44	0.436	0.145
*p*-value^a^	0.513	0.752		
p-adjust^a^	0.171	0.251		
**Triglycerides (mmol/L)**				
Baseline	2.33 ± 1.86	1.55 ± 0.71	0.147	0.049
End of trial	1.44 ± 1.38	1.43 ± 0.61		
Change^c^	-0.97 ± 0.81	–0.12 ± 0.99	0.022	0.007
*p*-value^a^	0.152	0.609		
p-adjust^a^	0.051	0.203		

^a^ Within-group differences; *p*-value is based on paired *t*-test. ^b^ Comparison between groups; *p*-value is based on two independent sample *t*-test. ^c^ Difference before and after the intervention. The mean difference is represented. The p-adjust less than 0.017 indicates a statistically significant result.

### 3.5 Safety measurements

Table 8 shows patient-reported adverse events throughout the study period. A total of 5 (17.24%) adverse events were recorded. All the adverse events that occurred in the Arg + fish oil group were related to the intakes of the Arg supplement, such as nausea, abdominal discomfort and diarrhea. Only two adverse events, abdominal discomfort and diarrhea occurred in the placebo group. However, there was a significant improvement after adjusting the dosage and frequency of the supplement. During the experiment, when this happens, we ask the patient to increase the number of times the supplement is consumed, from once a day to multiple times a day, or reduce the concentration by increasing the moisture.

**TABLE 8:** Patient-reported adverse events in the two groups.

Adverse events	Arg + fish oil (*n* = 14)	Placebo (*n* = 15)
Nausea	1	0
Abdominal discomfort	1	1
Dry mouth	0	0
Diarrhea	1	1
Total	3	2

Similarly, a safety assessment was conducted by blood biochemical markers of liver and kidney functions at the end of the trial (Table 8). It shows no significant difference between the Arg + fish oil group and placebo group at 12 weeks (Table 9).

**TABLE 9:** The blood biochemical markers of liver and kidney functions at the end of the trial.

Variables	Arg + fish oil (*n* = 14)	Placebo (*n* = 15)	t/z	*p*-value
Prealbumin, g/L	24.68 ± 3.94	23.69 ± 4.58	0.607	0.549
Lactate dehydrogenase, U/L	180.74 ± 76.1	198.20 ± 38.01	–0.323	0.747
Alkaline phosphatase, U/L	104.77 ± 46.91	92.30 ± 30.10	–0.184	0.854
γ-glutamyl transferase, U/L	53.93 ± 80.69	60.16 ± 81.40	–0.461	0.645
Globulin, g/L	29.43 ± 18.24	28.77 ± 6.03	–0.668	0.504
Albumin, g/L	40.61 ± 9.35	41.03 ± 2.75	–1.566	0.117
Total protein, g/L	67.84 ± 13.94	68.64 ± 6.93	–0.806	0.42
Indirect bilirubin, μmol/L	19.01 ± 18.99	10.03 ± 4.99	–1.198	0.231
Direct bilirubin, μmol/L	4.99 ± 2.23	3.69 ± 1.32	1.917	0.066
Total bilirubin, μmol/L	11.45 ± 5.92	10.97 ± 2.92	0.278	0.783
Alanine aminotransferase, U/L	21.31 ± 14.17	22.68 ± 20.01	–0.622	0.534
Uric acid, μmol/L	358.38 ± 116.22	309.20 ± 91.51	1.252	0.222
Creatinine, μmol/L	75.45 ± 22.78	75.40 ± 25.17	0.005	0.996
Carbamide, μmol/L	6.54 ± 1.56	6.65 ± 3.14	–0.125	0.902

## 4 Discussion

In this randomized controlled clinical trial in older adults with sarcopenia, we investigated for the first time the effects of arginine and fish oil supplementation on sarcopenia. Our findings demonstrate that arginine and fish oil significantly improved handgrip strength and gait speed in older adults with sarcopenia. Additionally, the Arg + fish oil group showed significant improvements in PASE and frailty status compared to the placebo group. Notably, at the end of the trial, TNF-α and IL-6 levels were significantly lower in the Arg + fish oil group than in the placebo group. These findings highlight the promising effects of arginine and fish oil supplementation in managing sarcopenia in older adults.

After 12 weeks of intervention, significant improvements in handgrip strength and gait speed were observed in the Arg + fish oil group compared to the placebo group. These findings suggest that the Arg and fish oil intervention may exert beneficial effects on muscle-related outcomes in older adults with sarcopenia. Our results align with previous studies demonstrating the positive impact of arginine on muscle health (31). Patricia et al. (32) reported that arginine-containing nutritional supplements enhance muscle strength and mass. Similarly, Børsheim et al. (33). found that supplementation with essential amino acids (EAAs) plus arginine improved lean body mass, muscle strength, and physical performance in elderly individuals with glucose intolerance. The underlying mechanisms may involve arginine’s metabolite, creatine, which activates the PI3K-Akt/PKB-mTOR pathway to promote muscle protein synthesis (34). Additionally, arginine enhances nitric oxide (NO) production, stimulates satellite cell proliferation, and restores skeletal muscle function (10).

Fish oil, rich in n-3 polyunsaturated fatty acids (PUFAs), particularly eicosapentaenoic acid (EPA) and docosahexaenoic acid (DHA), has also been linked to musculoskeletal health. Randomized controlled trials have demonstrated that n-3 PUFA supplementation can enhance muscle protein synthesis in older adults (35–37). Gordon et al. (38). supplemented older adults with 1.86 g EPA and 1.5 g DHA daily for 6 months and observed significant improvements in thigh muscle volume, handgrip strength, and one-repetition maximum (1-RM) muscle strength. Previous studies have also found that fish oil and curcumin can improve muscle mass, gait speed, and prevent age-related declines in muscle health in rats (39). These benefits may be attributed to n-3 PUFAs’ ability to activate the mTORC-1 signaling pathway, enhance protein translation, and inhibit muscle protein degradation, thereby improving muscle protein synthesis efficiency (40). Da Pan et al. found that the fish oil-derived ω-3 PUFA and wheat oligopeptide may enhance skeletal muscle strength by targeting the up-regulation of Tnnt1, Myosin, Fbln5 and Itgav (41).

However, this study found that there was no statistically significant change in SMI, suggesting that the improvement in sarcopenia may not be achieved through a direct increase in muscle mass. This seemingly contradictory result may stem from the fact that the 12-week intervention period may have focused more on inducing neuromuscular adaptations and metabolic improvements (42–44). This can enhance muscle strength and physical function, whereas structural muscle hypertrophy generally requires longer-term cumulative stimulation (45). Therefore, the synergistic effect of arginine and fish oil may have optimized the intrinsic functional properties of muscle without substantially altering its physical cross-sectional area in the short term.

In addition to muscle-related outcomes, our study also assessed the effects of arginine + fish oil supplementation on physical activity (PASE) and frailty in older adults. Frailty, a complex syndrome marked by decreased physiological reserve and multisystem dysfunction, is a strong predictor of negative outcomes like hospitalization and mortality (46). The improvements observed in PASE scores and frailty may be linked to the recovery of gait speed, as shown in previous studies (47). For example, Zhu et al. (48). found that nutritional supplements containing protein, β-hydroxy β-methylbutyrate, vitamin D, and omega-3 fatty acids improved physical activity scores in older adults. Similarly, Hsieh et al. (49). reported that combined home exercise and nutritional interventions had a positive effect on frailty and physical function in pre-frailty or frailty older adults.

However, there is currently less research on the effects of arginine and fish oil supplementation on skeletal muscle. The exact mechanisms behind the effects of arginine + fish oil supplementation on skeletal muscle remain unclear. Both arginine and fish oil are considered immunonutrients, with clinical studies showing their effectiveness in reducing inflammation and improving health outcomes in various populations. A prospective randomized study by Braga et al. found that (50) combinations of arginine and omega-3 fatty acids significantly reduced postoperative infections and hospital stays in surgical patients. Alexander further highlighted the anti-inflammatory and infection-preventing effects of combined arginine and fish oil supplementation (51). These findings suggest that the synergistic effects of arginine + fish oil may help reduce the harmful impact of inflammation on sarcopenia.

To elucidate the mechanisms, it is important to highlight the critical role of inflammation in the pathophysiology of sarcopenia. TNF-α and IL-6, as the main pro-inflammatory cytokines, play a key role in the process of muscle atrophy (52). TNF-α activates the NF-κB pathway, which not only mediates pro-inflammatory responses but also perpetuates a vicious cycle of cytokine overexpression (53). The TNF-α/NF-κB pro-inflammatory signaling pathway plays a dominant role in the mechanism of sarcopenia. This TNF-α also inhibits the Akt/mTOR signaling axis, leading to impaired protein synthesis and increased proteolysis in skeletal muscle (54, 55). Elevated levels of TNF-α or IL-6 can inhibit the expression of hormones such as growth hormone and IGF-1 (56, 57). This leads to impaired muscle production and increased proteolysis, which together promote structural destruction and functional decline of muscles (58). Studies have shown that arginine can increase the production of NO and inhibit the activation of the NF-κB signaling pathway, thereby effectively reducing the expression of TNF-α and IL-6 (59). In this study, we also demonstrated that arginine and fish oil supplementation significantly reduced TNF-α and IL-6 levels in patients with sarcopenia. This is consistent with previous studies in which Irandoust (60) et al. found a significant decrease in plasma IL-6 following arginine supplementation in trained men. Similarly, n-3 PUFAs in fish oil have been shown to reduce pro-inflammatory cytokines while increasing anti-inflammatory factors like IL-10 and TGF-β (11). Multiple studies have also demonstrated the effects of arginine and fish oil in reducing IL-6 and TNF-α (61–63). In addition, both arginine and fish oil can stimulate the mTOR signaling pathway and increase the rate of muscle protein synthesis, thereby counteracting the inhibitory effect of inflammation on mTOR. Therefore, we speculate that arginine and fish oil can improve the symptoms of sarcopenia and delay the progression by reducing IL-6 and TNF-α.

However, our study did not observe significant improvements in muscle mass, possibly due to the complex pathogenesis of sarcopenia (64–66). Factors such as malnutrition, physical inactivity, aging, chronic comorbidities, and hormonal changes contribute to inflammation and malabsorption, all of which can exacerbate muscle loss and dysfunction. Aging is known to reduce the sensitivity of skeletal muscle protein synthesis to dietary amino acids, and older adults need more dietary proteins and essential amino acids to stimulate the same rate of synthesis as younger adults (67). Therefore, optimizing protein intake or supplementing with amino acids for specific functions has been used as a basis for the prevention and management of muscle loss in patients with sarcopenia. In our study, completers exhibited stable muscle function and strength parameters during the continuation of the trial. This suggests that while supplementing with function-specific amino acids does not improve muscle mass, it may reduce the natural progression of muscle dysfunction and muscle loss.

The advantages of our research are as follows. First, our results were obtained in older adults with sarcopenia, as few studies have focused specifically on the effects of arginine and fish oil in this population. Second, we observed the additive effects of RET-based arginine and fish oil on sarcopenia. However, our study has some limitations. First, the control group did not receive placebo capsules that were visually identical to the fish oil capsules. Although all assessors performing measurements for the objective outcome measures were blinded to group allocation, and all primary outcomes were objective indicators, the trial design did not achieve perfect double-blinding at the participant level, theoretically introducing the potential for participant expectation bias. Second, the mechanistic discussion above is primarily based on inferences drawn from existing literature, as this study did not directly measure relevant molecular biomarkers. Therefore, the conclusions presented should be regarded as hypotheses regarding potential mechanisms underlying the clinical outcomes. Third, the small sample size may have limited statistical power, making it difficult to detect significant differences in certain parameters. Future studies should involve larger cohorts to enhance the robustness and generalizability of the findings. Fourth, the 12-week intervention period cannot answer the potential effect of interventions on a long-term perspective. Fifth, the absence of separate component intervention groups limits our ability to dissect the individual contributions of each constituent to the combined effect. Future research should utilize placebos that are perfectly matched in dosage form, quantity, and method of administration, implement a multi-group design, extend the intervention period, and incorporate related mechanistic studies to validate and expand upon the findings of this study.

## 5 Conclusion

In conclusion, combined supplementation with arginine and fish oil improves muscle strength, gait speed, and inflammatory status in older adults with sarcopenia. These findings suggest that Arg + fish oil may serve as a promising therapeutic strategy for sarcopenia management. However, further large-scale, multicenter studies are needed to confirm these results and explore the long-term benefits of this intervention.

## Data Availability

The original contributions presented in the study are included in the article/Supplementary material, further inquiries can be directed to the corresponding author.

## References

[1] Ogawa S. [Clinical manifestation and pathophyisological bases of sarcopenia]. *Clin Calcium*. (2016) 26:1703–8. 27885181

[2] Chen L Woo J Assantachai P Auyeung T Chou M Iijima K Asian working group for Sarcopenia: 2019 consensus update on sarcopenia diagnosis and treatment. *J Am Med Dir Assoc*. (2020) 21:300–07.e2. 10.1016/j.jamda.2019.12.012 32033882

[3] Gordon B Kelleher A Kimball S. Regulation of muscle protein synthesis and the effects of catabolic states. *Int J Biochem Cell Biol*. (2013) 45:2147–57. 10.1016/j.biocel.2013.05.039 23769967 PMC3759561

[4] Li C Yu K Shyh-Chang N Li G Jiang L Yu S Circulating factors associated with sarcopenia during ageing and after intensive lifestyle intervention. *J Cachexia Sarcopenia Muscle*. (2019) 10:586–600. 10.1002/jcsm.12417 30969486 PMC6596393

[5] Li W Yue T Liu Y. New understanding of the pathogenesis and treatment of stroke-related sarcopenia. *Biomed Pharmacother*. (2020) 131:110721. 10.1016/j.biopha.2020.110721 32920517

[6] Papadopoulou S. Sarcopenia: a contemporary health problem among older adult populations. *Nutrients*. (2020) 12:1293. 10.3390/nu12051293 32370051 PMC7282252

[7] Xie W He M Yu D Wu Y Wang X Lv S Mouse models of sarcopenia: classification and evaluation. *J Cachexia Sarcopenia Muscle*. (2021) 12:538–54. 10.1002/jcsm.12709 33951340 PMC8200444

[8] Cho M Lee S Song SK. A review of sarcopenia pathophysiology, diagnosis, treatment and future direction. *J Korean Med Sci*. (2022) 37:e146. 10.3346/jkms.2022.37.e146 35535373 PMC9091430

[9] Chilibeck P Kaviani M Candow D Zello G. Effect of creatine supplementation during resistance training on lean tissue mass and muscular strength in older adults: a meta-analysis. *Open Access J Sports Med*. (2017) 8:213–26. 10.2147/OAJSM.S123529 29138605 PMC5679696

[10] Buono R Vantaggiato C Pisa V Azzoni E Bassi M Brunelli S Nitric oxide sustains long-term skeletal muscle regeneration by regulating fate of satellite cells via signaling pathways requiring Vangl2 and cyclic GMP. *Stem Cells*. (2011) 30:197–209. 10.1002/stem.783 22084027 PMC3378700

[11] Hui-lan G Yan-yu L Xiao-xu H Pian-hong Z. Effects of N-3 polyunsaturated fatty acids on Sarcopenia in the elderly: a narrative review. *Acta Nutrimenta Sinica.* (2021) 43:302–6. 10.13325/j.cnki.acta.nutr.sin.2021.03.015

[12] Xu D Lu Y Yang X Pan D Wang Y Yin S Effects of fish oil-derived n-3 polyunsaturated fatty acid on body composition, muscle strength and physical performance in older people: a secondary analysis of a randomised, double-blind, placebo-controlled trial. *Age Ageing*. (2022) 51:afac274. 10.1093/ageing/afac274 36571774

[13] Uchida Y Tsuji K Ochi E. Effects of Omega-3 fatty acids supplementation and resistance training on skeletal muscle. *Clin Nutr ESPEN*. (2024) 61:189–96. 10.1016/j.clnesp.2024.03.019 38777432

[14] Therdyothin A Phiphopthatsanee N Isanejad M. The effect of omega-3 fatty acids on Sarcopenia: mechanism of action and potential efficacy. *Mar Drugs*. (2023) 21:399. 10.3390/md21070399 37504930 PMC10381755

[15] Jost Z Tomczyk M Chroboczek M Calder P Fisk H Przewłócka K Increased plasma L-arginine Levels and L-arginine/ADMA ratios after twelve weeks of omega-3 fatty acid supplementation in amateur male endurance runners. *Nutrients*. (2022) 14:4749. 10.3390/nu14224749 36432437 PMC9699131

[16] Toupchian O Sotoudeh G Mansoori A Nasli-Esfahani E Djalali M Keshavarz S Effects of DHA-enriched fish oil on monocyte/macrophage activation marker sCD163, asymmetric dimethyl arginine, and insulin resistance in type 2 diabetic patients. *J Clin Lipidol*. (2016) 10:798–807. 10.1016/j.jacl.2016.02.013 27578110

[17] Ma C Tsai H Su W Sun L Shih Y Wang J. Combination of arginine, glutamine, and omega-3 fatty acid supplements for perioperative enteral nutrition in surgical patients with gastric adenocarcinoma or gastrointestinal stromal tumor (GIST): a prospective, randomized, double-blind study. *J Postgrad Med*. (2018) 64:155–63. 10.4103/jpgm.JPGM_693_17 29848836 PMC6066627

[18] Mazaki T Ishii Y Murai I. Immunoenhancing enteral and parenteral nutrition for gastrointestinal surgery: a multiple-treatments meta-analysis. *Ann Surg*. (2014) 261:662–9. 10.1097/SLA.0000000000000935 25405556

[19] Drover J Dhaliwal R Weitzel L Wischmeyer P Ochoa J Heyland D. Perioperative use of arginine-supplemented diets: a systematic review of the evidence. *J Am Coll Surg*. (2011) 212:385–99.e1. 10.1016/j.jamcollsurg.2010.10.016 21247782

[20] Marimuthu K Varadhan K Ljungqvist O Lobo DN. A meta-analysis of the effect of combinations of immune modulating nutrients on outcome in patients undergoing major open gastrointestinal surgery. *Ann Surg*. (2012) 255:1060–8. 10.1097/SLA.0b013e318252edf8 22549749

[21] Miller L Douglas C McCullough F Stanworth S Calder P. Impact of enteral immunonutrition on infectious complications and immune and inflammatory markers in cancer patients undergoing chemotherapy: a systematic review of randomised controlled trials. *Clin Nutr*. (2022) 41:2135–46. 10.1016/j.clnu.2022.07.039 36067585

[22] Morris S. Arginine metabolism revisited. *J Nutr*. (2016) 146:2579S–86S. 10.3945/jn.115.226621 27934648

[23] Tong B Barbul A. Cellular and physiological effects of arginine. *Mini Rev Med Chem*. (2004) 4:823–32. 10.2174/1389557043403305 15544543

[24] Li S Lu Z Leung J Kwok T. Association of dietary protein intake, inflammation with muscle mass, physical performance and incident sarcopenia in Chinese community-dwelling older adults. *J Nutr Health Aging*. (2024) 28:100163. 10.1016/j.jnha.2024.100163 38350300 PMC12877208

[25] Hatami O Aghabagheri M Kahdouei S Nasiriani K. Psychometric properties of the Persian version of the physical activity scale for the elderly (PASE). *BMC Geriatr*. (2021) 21:383. 10.1186/s12877-021-02337-0 34162345 PMC8220717

[26] Ng Y Cheng L Quek Y Yu R Wu X. The measurement properties and feasibility of FRAIL scale in older adults: a systematic review and meta-analysis. *Ageing Res Rev*. (2024) 95:102243. 10.1016/j.arr.2024.102243 38395198

[27] Zitser J Allen I Falgàs N Le M Neylan T Kramer J Pittsburgh sleep quality index (PSQI) responses are modulated by total sleep time and wake after sleep onset in healthy older adults. *PLoS One*. (2022) 17:e0270095. 10.1371/journal.pone.0270095 35749529 PMC9232154

[28] Trampisch U Pourhassan M Daubert D Volkert D Wirth R. Interrater reliability of routine screening for risk of malnutrition with the mini nutritional assessment short-form in hospital. *Eur J Clin Nutr*. (2022) 76:1111–6. 10.1038/s41430-022-01080-y 35194196 PMC9352578

[29] Tu Y Chen F Yu Q Song L Chen M. Application of NRS2002 and PG-SGA in nutritional assessment for perioperative patients with head and neck squamous cell carcinoma: an observational study. *Medicine.* (2024) 103:e40025. 10.1097/MD.0000000000040025 39470500 PMC11521053

[30] Jian G Jia-qing F Li-jing J Wen-qian Y Bin L Hong-wei G. Assessment of the reproducibility and validity of a simple food-frequency questionnaire used in dietary patterns studies. *Acta Nutrimenta Sinica.* (2011) 33:452–6. 10.13325/j.cnki.acta.nutr.sin.2011.05.012

[31] Córdova-Martínez A Caballero-García A Bello H Pons-Biescas A Noriega D Roche E. l-Arginine and beetroot extract supplementation in the prevention of Sarcopenia. *Pharmaceuticals.* (2022) 15:290. 10.3390/ph15030290 35337088 PMC8954952

[32] May P Barber A D’Olimpio J Hourihane A Abumrad N. Reversal of cancer-related wasting using oral supplementation with a combination of beta-hydroxy-beta-methylbutyrate, arginine, and glutamine. *Am J Surg*. (2002) 183:471–9. 10.1016/s0002-9610(02)00823-1 11975938

[33] Børsheim E Bui Q Tissier S Kobayashi H Ferrando A Wolfe R. Effect of amino acid supplementation on muscle mass, strength and physical function in elderly. *Clin Nutr*. (2008) 27:189–95. 10.1016/j.clnu.2008.01.001 18294740 PMC2430042

[34] Deldicque L Louis M Theisen D Nielens H Dehoux M Thissen J Increased IGF mRNA in human skeletal muscle after creatine supplementation. *Med Sci Sports Exerc*. (2005) 37:731–6. 10.1249/01.mss.0000162690.39830.27 15870625

[35] Murphy C Flanagan E De Vito G Susta D Mitchelson K de Marco Castro E Does supplementation with leucine-enriched protein alone and in combination with fish-oil-derived n-3 PUFA affect muscle mass, strength, physical performance, and muscle protein synthesis in well-nourished older adults? A randomized, double-blind, placebo-controlled trial. *Am J Clin Nutr*. (2021) 113:1411–27. 10.1093/ajcn/nqaa449 33871558 PMC8168361

[36] Brook M Din U Tarum J Selby A Quinlan J Bass J Omega-3 supplementation during unilateral resistance exercise training in older women: a within subject and double-blind placebo-controlled trial. *Clin Nutr ESPEN*. (2021) 46:394–404. 10.1016/j.clnesp.2021.09.729 34857226 PMC8629763

[37] Ma W Li H Zhang W Zhai J Li J Liu H Effect of n-3 polyunsaturated fatty acid supplementation on muscle mass and function with aging: a meta-analysis of randomized controlled trials?. *Prostaglandins Leukot Essent Fatty Acids*. (2021) 165:102249. 10.1016/j.plefa.2021.102249 33485255

[38] Smith G Julliand S Reeds D Sinacore D Klein S Mittendorfer B. Fish oil-derived n-3 PUFA therapy increases muscle mass and function in healthy older adults. *Am J Clin Nutr*. (2015) 102:115–22. 10.3945/ajcn.114.105833 25994567 PMC4480667

[39] Kuszewski J Wong R Wood L Howe P. Effects of fish oil and curcumin supplementation on cerebrovascular function in older adults: a randomized controlled trial. *Nutr Metab Cardiovasc Dis*. (2020) 30:625–33. 10.1016/j.numecd.2019.12.010 32127335

[40] López-Seoane J Jiménez S Del Coso J Pareja-Galeano H. Muscle hypertrophy induced by N-3 PUFA supplementation in absence of exercise: a systematic review of randomized controlled trials. *Crit Rev Food Sci Nutr*. (2023) 63:6536–46. 10.1080/10408398.2022.2034734 35112608

[41] Rondanelli M Gasparri C Barrile G Battaglia S Cavioni A Giusti R Effectiveness of a novel food composed of leucine, omega-3 fatty acids and probiotic Lactobacillus paracasei PS23 for the treatment of Sarcopenia in elderly subjects: a 2-month randomized double-blind placebo-controlled trial. *Nutrients*. (2022) 14:4566. 10.3390/nu14214566 36364828 PMC9656258

[42] Lee S Nam W An K Cho E Choi Y Ryu HY. L-arginine supplementation improves endurance under chronic fatigue: inducing in vivo paradigms with in vitro support. *Nutrients*. (2025) 17:3239. 10.3390/nu17203239 41156492 PMC12566733

[43] McGlory C Calder P Nunes E. The influence of omega-3 fatty acids on skeletal muscle protein turnover in health. *Disuse, and Disease. Front Nutr*. (2019) 6:144. 10.3389/fnut.2019.00144 31555658 PMC6742725

[44] Tseng P Zeng B Zeng B Liao Y Stubbs B Kuo J Omega-3 polyunsaturated fatty acids in sarcopenia management: a network meta-analysis of randomized controlled trials. *Ageing Res Rev*. (2023) 90:102014. 10.1016/j.arr.2023.102014 37442370

[45] Seynnes O de Boer M Narici M. Early skeletal muscle hypertrophy and architectural changes in response to high-intensity resistance training. *J Appl Physiol.* (2006) 102:368–73. 10.1152/japplphysiol.00789.2006 17053104

[46] Hoogendijk E Afilalo J Ensrud K Kowal P Onder G Fried L. Frailty: implications for clinical practice and public health. *Lancet*. (2019) 394:1365–75. 10.1016/S0140-6736(19)31786-6 31609228

[47] World Health Organization. *Who Guidelines Approved by the Guidelines Review Committee. Who Guidelines on Physical Activity and Sedentary Behaviour.* Geneva: World Health Organization (2020).

[48] Zhu L Chan R Kwok T Cheng K Ha A Woo J. Effects of exercise and nutrition supplementation in community-dwelling older Chinese people with sarcopenia: a randomized controlled trial. *Age Ageing*. (2019) 48:220–8. 10.1093/ageing/afy179 30462162

[49] Hsieh T Su S Chen C Kang Y Hu M Hsu L Individualized home-based exercise and nutrition interventions improve frailty in older adults: a randomized controlled trial. *Int J Behav Nutr Phys Act*. (2019) 16:119. 10.1186/s12966-019-0855-9 31791364 PMC6889427

[50] Braga M Gianotti L Nespoli L Radaelli G Di Carlo V. Nutritional approach in malnourished surgical patients: a prospective randomized study. *Arch Surg*. (2002) 137:174–80. 10.1001/archsurg.137.2.174 11822956

[51] Alexander J Supp D. Role of arginine and omega-3 fatty acids in wound healing and infection. *Adv Wound Care.* (2014) 3:682–90. 10.1089/wound.2013.0469 25371851 PMC4217020

[52] Byrne T Cooke J Bambrick P McNeela E Harrison M. Circulating inflammatory biomarker responses in intervention trials in frail and sarcopenic older adults: a systematic review and meta-analysis. *Exp Gerontol*. (2023) 177:112199. 10.1016/j.exger.2023.112199 37156445

[53] Glass D. Signaling pathways perturbing muscle mass. *Curr Opin Clin Nutr Metab Care*. (2010) 13:225–9. 10.1097/mco.0b013e32833862df 20397318

[54] Li H Malhotra S Kumar A. Nuclear factor-kappa B signaling in skeletal muscle atrophy. *J Mol Med.* (2008) 86:1113–26. 10.1007/s00109-008-0373-8 18574572 PMC2597184

[55] Dupont J Vercauteren L Amini N Lapauw L De Schaepdryver M Poesen K Are inflammatory markers associated with sarcopenia-related traits in older adults with sarcopenia? - A cross-sectional analysis of the ENHANce study. *Exp Gerontol*. (2023) 178:112196. 10.1016/j.exger.2023.112196 37156446

[56] Fernández-Celemín L Pasko N Blomart V Thissen J. Inhibition of muscle insulin-like growth factor I expression by tumor necrosis factor-alpha. *Am J Physiol Endocrinol Metab*. (2002) 283:E1279–90. 10.1152/ajpendo.00054.2002 12388149

[57] Foulstone E Meadows K Holly J Stewart C. Insulin-like growth factors (IGF-I and IGF-II) inhibit C2 skeletal myoblast differentiation and enhance TNF alpha-induced apoptosis. *J Cell Physiol*. (2001) 189:207–15. 10.1002/jcp.10017 11598906

[58] Pototschnig I Feiler U Diwoky C Vesely P Rauchenwald T Paar M Interleukin-6 initiates muscle- and adipose tissue wasting in a novel C57BL/6 model of cancer-associated cachexia. *J Cachexia Sarcopenia Muscle*. (2023) 14:93–107. 10.1002/jcsm.13109 36351437 PMC9891934

[59] Martí I Líndez AA Reith W. Arginine-dependent immune responses. *Cell Mol Life Sci*. (2021) 78:5303–24. 10.1007/s00018-021-03828-4 34037806 PMC8257534

[60] IrandoustK HamzehlooA YouzbashiL TaheriM Ben SaadH. High intensity interval training and L-Arginine supplementation decrease interleukin-6 levels in adult trained males. *Tunis Med*. (2022) 100:696–705.36571754

[61] Gianotti L Braga M Fortis C Soldini L Vignali A Colombo S A prospective, randomized clinical trial on perioperative feeding with an arginine-, omega-3 fatty acid-, and RNA-enriched enteral diet: effect on host response and nutritional status. *JPEN J Parenter Enteral Nutr*. (1999) 23:314–20. 10.1177/0148607199023006314 10574478

[62] Guo Z Ge C Han B Qie Z Kang Y Zhu Y Effects of parenteral nutrition of Ω-3 polyunsaturated fatty acid, arginine and glutamine on cellular immune status of patients following liver cancer surgery. *Trop J Pharm Res.* (2018) 17:507–11.

[63] Blok W Deslypere J Demacker P van der Ven-Jongekrijg J Hectors M van der Meer J Pro- and anti-inflammatory cytokines in healthy volunteers fed various doses of fish oil for 1 year. *Eur J Clin Invest*. (1997) 27:1003–8. 10.1046/j.1365-2362.1997.2240775.x 9466128

[64] Cao W Zhu A Chu S Zhou Q Zhou Y Qu X Correlation between nutrition, oral health, and different sarcopenia groups among elderly outpatients of community hospitals: a cross-sectional study of 1505 participants in China. *BMC Geriatr*. (2022) 22:332. 10.1186/s12877-022-02934-7 35428189 PMC9013090

[65] Wunderle C Haller L Laager R Bernasconi L Neyer P Stumpf F The association of the essential amino acids lysine, methionine, and threonine with clinical outcomes in patients at nutritional risk: secondary analysis of a randomized clinical trial. *Nutrients* (2024) 16:2608. 10.3390/nu16162608 39203745 PMC11357570

[66] König M Spira D Demuth I Steinhagen-Thiessen E Norman K. Polypharmacy as a risk factor for clinically relevant Sarcopenia: results from the berlin aging study II. *J Gerontol A Biol Sci Med Sci*. (2017) 73:117–22. 10.1093/gerona/glx074 28481965

[67] Brack A Muñoz-Cánoves P. The ins and outs of muscle stem cell aging. *Skelet Muscle*. (2016) 6:1. 10.1186/s13395-016-0072-z 26783424 PMC4716636

