# Supplementary figures and images for: Twelve-week combined arginine and fish oil supplementation is associated with reduced sarcopenia severity: a randomized, double-blind, placebo-controlled study
Source: Front Nutr. 2026 Mar 2;13:1763219. doi: 10.3389/fnut.2026.1763219 (PMC12989373; doi:10.3389/fnut.2026.1763219)

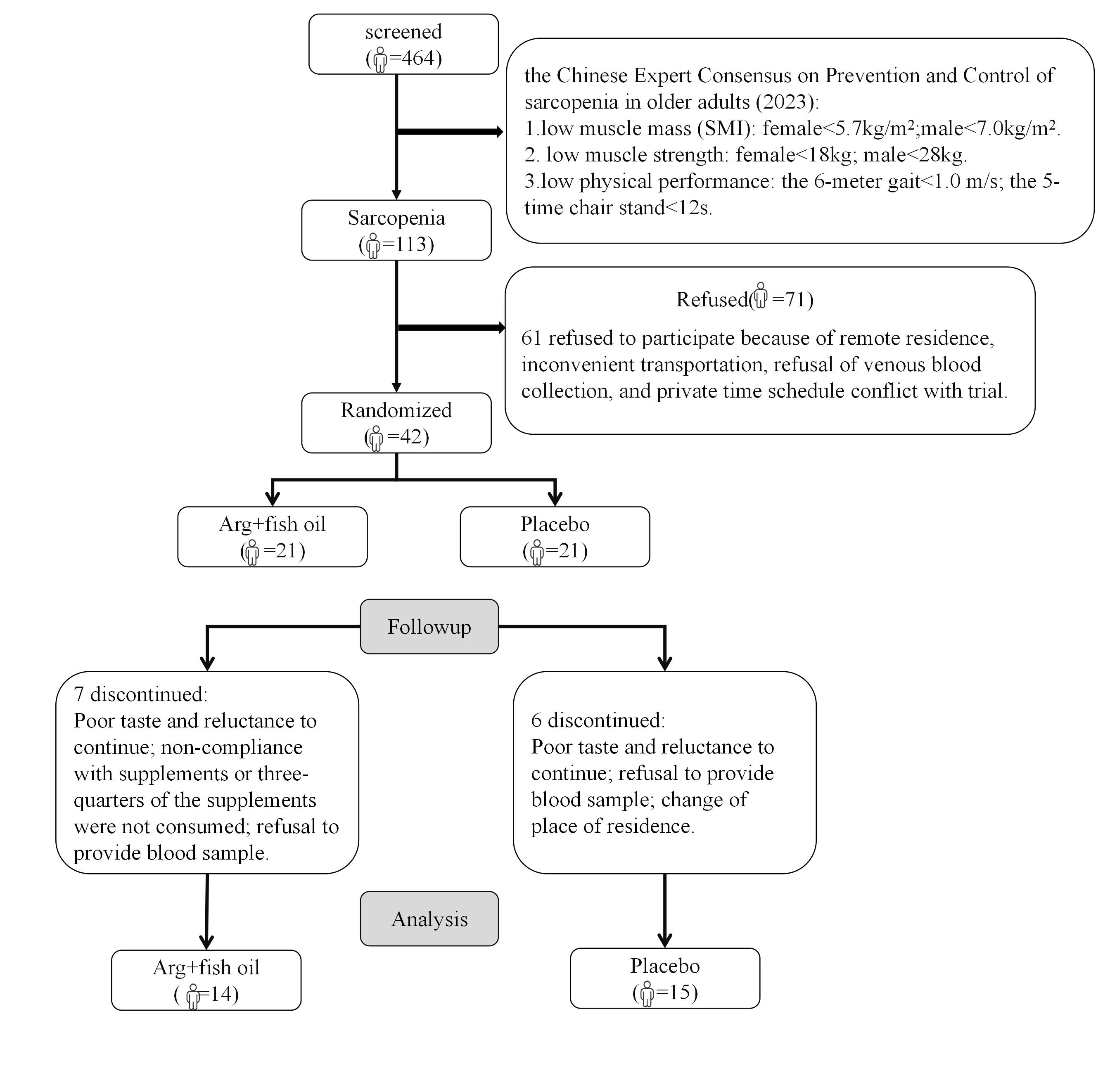

Supplement: Supplementary file 1 [file Image_1.jpeg]
